# Efficacy and safety of Oliceridine versus Sufentanil in postoperative analgesia for burn skin grafting: a machine learning and SHAP-based cohort study

**DOI:** 10.3389/fmed.2026.1821562

**Published:** 2026-06-09

**Authors:** Ye Tian, Yun Zhang, Danshi Feng, Hongying Wang, Zihuan Ma

**Affiliations:** 1 Department of Anesthesiology, Pangang Group General Hospital, Panzhihua, Sichuan, China; 2 School of Anesthesiology, Zunyi Medical University, Zunyi, Guizhou, China

**Keywords:** machine learning, Oliceridine, postoperative nausea and vomiting, SHAP interpretability, Sufentanil

## Abstract

**Background:**

Balancing profound analgesia with the minimization of opioid-related adverse events, particularly postoperative nausea and vomiting (PONV), remains challenging in burn surgery. This study aimed to use machine learning to evaluate the comparative efficacy and safety of the novel biased agonist Oliceridine versus Sufentanil.

**Methods:**

A retrospective cohort of 586 patients undergoing burn skin grafting was analyzed. XGBoost algorithms were developed to predict the need for rescue analgesia (efficacy) and PONV occurrence (safety). The Shapley additive explanations (SHAP) were applied to decipher feature contributions and drug effects.

**Results:**

The XGBoost efficacy model (AUC = 0.843) identified total burn surface area and surgery duration as dominant predictors, whereas opioid choice had negligible SHAP impact, suggesting a comparable predictive analgesic profile between Oliceridine and Sufentanil in this cohort. Conversely, the XGBoost PONV model (AUC = 0.788) outperformed logistic regression, identifying Sufentanil administration and female patients as paramount risk drivers. SHAP interaction analysis revealed a strong predictive association in which substituting Sufentanil with Oliceridine correlated with a substantially blunted predicted PONV risk trajectory in female patients.

**Conclusion:**

Machine learning evaluation suggests a distinction between opioid predicted efficacy and safety profiles in this cohort. Oliceridine showed similar predicted analgesic outcomes to Sufentanil while being associated with a significantly lower predicted risk of PONV. These exploratory insights provide hypothesis-generating frameworks for risk stratification in severe burn management, though further prospective trials are required to establish any clinical validity.

## 1 Introduction

Severe burn injuries represent a substantial global public health burden, with persistent disparities in clinical outcomes underscoring an urgent need for optimized and targeted therapeutic interventions (1). Furthermore, subsequent skin grafting procedures inflict excruciating nociceptive and neuropathic pain, necessitating aggressive, opioid-based perioperative analgesia (2–4). However, the administration of traditional high-potency opioids, i.e., Sufentanil, frequently provokes opioid-related adverse events (ORAEs) (5, 6). Postoperative nausea and vomiting (PONV) is one of them, and it is highly debilitating, contributing to delayed recovery, the risk of aspiration, and even to the physical disturbance of the delicate skin grafts (7, 8). Accordingly, striking a subtle balance between deep nociceptive inhibition and the reduction of ORAEs remains an ongoing anesthetic challenge in burn care (9).

Oliceridine is a new pharmacological concept which is a G-protein signaling-selective (biased) *μ*-agonist of μ-opioid receptors (10, 11). Unlike the classical opioids that selectively bind the G-protein signaling (leading to analgesia) and the *β*-arrestin pathway (inducing respiratory depression and gastrointestinal dysfunction), Oliceridine selectively evokes the G-protein signaling (12, 13). Although preliminary clinical trials in general surgical populations have shown that it exhibits comparable analgesic effects to morphine and demonstrates a more favorable overall safety profile, there is a considerable dearth of empirical evidence evaluating its comparative efficacy and safety in patients under extreme physiological stress, such as major burn patients (14).

Furthermore, individual patient responses to particular opioid regimens are inherently complex and multifactorial (15). Traditional linear statistical models frequently fail to identify the high-order, non-linear interactions among demographic variables, the severity of surgical trauma, and pharmacological interventions (16). Machine learning (ML) algorithms, particularly gradient boosting models such as eXtreme Gradient Boosting (XGBoost), have demonstrated superior discriminative strength in modeling complex clinical outcomes (17, 18). However, the problem lies in the fact that the advanced ML models are not easily integrated into clinical practice because of their black-box nature (19). The application of Shapley additive explanations (SHAP) is a method to overcome this translational challenge by offering mathematically rigorous, instance-based transparency, elucidating the precise mechanisms and feature contributions behind individualized predictions (20).

Therefore, this retrospective study aimed to construct and validate robust ML models to predict both postoperative analgesic efficacy (need for rescue analgesia) and safety (incidence of PONV) in burn skin grafting patients using Oliceridine or Sufentanil. This study aimed to agnostically decipher the underlying feature contributions of opioid selection and ultimately present the algorithmic findings as exploratory mathematical models to generate hypotheses regarding personalized perioperative pain management.

## 2 Materials and methods

### 2.1 Study design and ethical considerations

This study was conducted as a single-center, retrospective observational cohort analysis to evaluate the comparative efficacy and safety of Oliceridine versus Sufentanil for postoperative analgesia in patients undergoing burn skin grafting. The study protocol, along with the feasibility report and informed consent forms, was comprehensively reviewed and approved by the Institutional Review Board (IRB) and the Clinical Trial Ethics Committee of Pangang Group General Hospital (Approval Number: NOPGZYY-LLKY-2024017). The study was executed in strict accordance with the ethical principles outlined in the Declaration of Helsinki and the Good Clinical Practice (GCP) guidelines issued by the National Medical Products Administration (NMPA) of China. Given the retrospective nature of the electronic medical record analysis, written informed consent was waived by the IRB; however, all patient data were meticulously de-identified and anonymized prior to extraction and analysis to ensure strict patient confidentiality.

### 2.2 Study population and eligibility criteria

The study cohort consisted of adult patients who underwent elective or emergency burn skin grafting surgery at the Pangang Group General Hospital. The primary inclusion criteria included: (1) age between 18 and 80 years; (2) American Society of Anesthesiologists (ASA) physical status I–III; (3) scheduled for burn debridement and skin grafting under general anesthesia; and (4) continuous postoperative patient-controlled intravenous analgesia (PCIA) using either Oliceridine or Sufentanil as the primary opioid analgesic. The institutional standardized PCIA protocol included a background infusion with on-demand bolus doses and a 15-min lockout interval, supplemented by routine prophylactic antiemetics at the anesthesiologist’s discretion prior to extubation. Patients were excluded when they met any of the following criteria: (1) documented allergy or hypersensitivity to any study medications; (2) preoperative history of chronic opioid dependence, substance abuse, or severe psychiatric disorders; (3) significant hepatic or renal dysfunction precluding standard opioid metabolism; (4) pregnancy or active lactation; or (5) incomplete medical records regarding key perioperative parameters or targeted clinical outcomes.

### 2.3 Data extraction and feature definition

A comprehensive dataset encompassing patient demographics, burn characteristics, intraoperative variables, and postoperative outcomes was extracted from the electronic health record (EHR) and anesthesia information management systems of the hospital. To build the ML models, 11 raw clinical variables were predetermined. Their selection was strictly guided by established clinical expertise and *a priori* domain knowledge regarding pain management and opioid-related side effects, rather than automated linear feature-selection algorithms (such as univariable *p*-value screening; or least absolute shrinkage and selection operator, LASSO regression). This deliberate exclusion of linear pre-filtering ensured that variables with potentially weak independent effects but strong non-linear or synergistic interactions were preserved for the tree-based ML framework. Furthermore, considering the incidence of the minority class events in the study cohort (approximately 178 occurrences of PONV), the inclusion of 15 final engineered features yielded an events per variable (EPV) ratio of approximately 11.8, comfortably exceeding the universally recommended safety threshold of 10 to effectively minimize the risk of model overfitting.

The extracted continuous variables consisted of age (years), body mass index (BMI, kg/m^2^), total body surface area (TBSA) burned (%), third-degree burn area (%), baseline mean arterial pressure (MAP, mmHg), baseline heart rate (HR, beats/min), duration of surgery (min), and intraoperative blood loss (mL). The categorical variables included gender (men or women), ASA physical status (I/II/III), and treatment group (Oliceridine or Sufentanil). During data preprocessing, these three categorical variables were transformed using one-hot encoding, resulting in a final set of 15 distinct input features for model training.

### 2.4 Clinical outcomes

The study defined two primary endpoints to evaluate the dual facets of postoperative care.

Analgesic efficacy outcome: Defined as a binary classification (Yes/No) indicating whether a patient required rescue analgesia within 48 h postoperatively due to inadequate pain control through the continuous PCIA protocol. Additional descriptive efficacy metrics included the numeric rating scale (NRS, 0–10 points) pain score at 24 h post-surgery and the exact time (min) to the first administration of rescue analgesia among event-positive patients.

Safety outcome: Defined as a binary classification (Yes/No) indicating the occurrence of clinically significant PONV requiring pharmacological intervention within the initial 48-h postoperative period.

### 2.5 Machine learning model development

Model development and validation were conducted in strict adherence to the transparent reporting of a multivariable prediction model for individual prognosis or diagnosis (TRIPOD) statement. To predict the requirement for rescue analgesia and the occurrence of PONV, this study evaluated four distinct ML classification algorithms: logistic regression (LR), support vector machine (SVM) using a radial basis function kernel, random forest (RF), and eXtreme Gradient Boosting (XGBoost). Missing data were not observed in the final analytical cohort. The total cohort was partitioned into a 70% training set and a 30% independent hold-out test set using a stratified random splitting strategy to preserve the proportional distribution of the outcome classes, thereby inherently managing the moderate class imbalance without the need for synthetic resampling techniques such as synthetic minority over-sampling technique (SMOTE). Prior to model training, categorical variables were one-hot encoded, and continuous features were standardized using *Z*-score normalization specifically for scale-sensitive algorithms (LR and SVM). Hyperparameter optimization was conducted exclusively within the training set using a five-fold cross-validation grid search approach, guided by maximization of the area under the curve (AUC). For the final XGBoost model, the tuned parameters included a maximum tree depth of 5, a learning rate of 0.03, subsample and colsample_bytree ratios of 0.8, and a minimum child weight of 2 to strictly prevent overfitting. Model performance on the unseen test set was systematically assessed using AUC with 95% confidence intervals, precision-recall (PR) curves, Brier scores for formal calibration quantification, and confusion matrices. Additionally, decision curve analysis (DCA) was performed to evaluate the clinical usefulness of the predictive models.

### 2.6 SHAP interpretability and clinical translation

The SHAP framework was used to demystify the internal decision making architecture of the optimal ML model (XGBoost) and remove the phenomenon of the black box. In particular, the TreeSHAP algorithm was applied to obtain specific feature attributions for individual predictions. Beeswarm plots and mean absolute SHAP value bar graphs were used to visualize the interpretability of global models. Non-linear relationships and interaction effects among features (specifically the interplay between opioid selection, patient gender, and burn severity) were examined through SHAP dependence plots. Moreover, instance-level clinical reasoning was also shown in SHAP waterfall plots of high- and low-risk patient cases. Finally, partial dependence plots (PDP), predictive risk stratification tertiles, and multivariable odds ratio forest plots were generated to translate these algorithmic insights into actionable clinical guidelines. All statistical calculations and ML were performed in R programming language (4.4.0, R foundation to Statistical computing).

## 3 Results

### 3.1 Baseline characteristics and clinical outcomes

**Figure 1:**
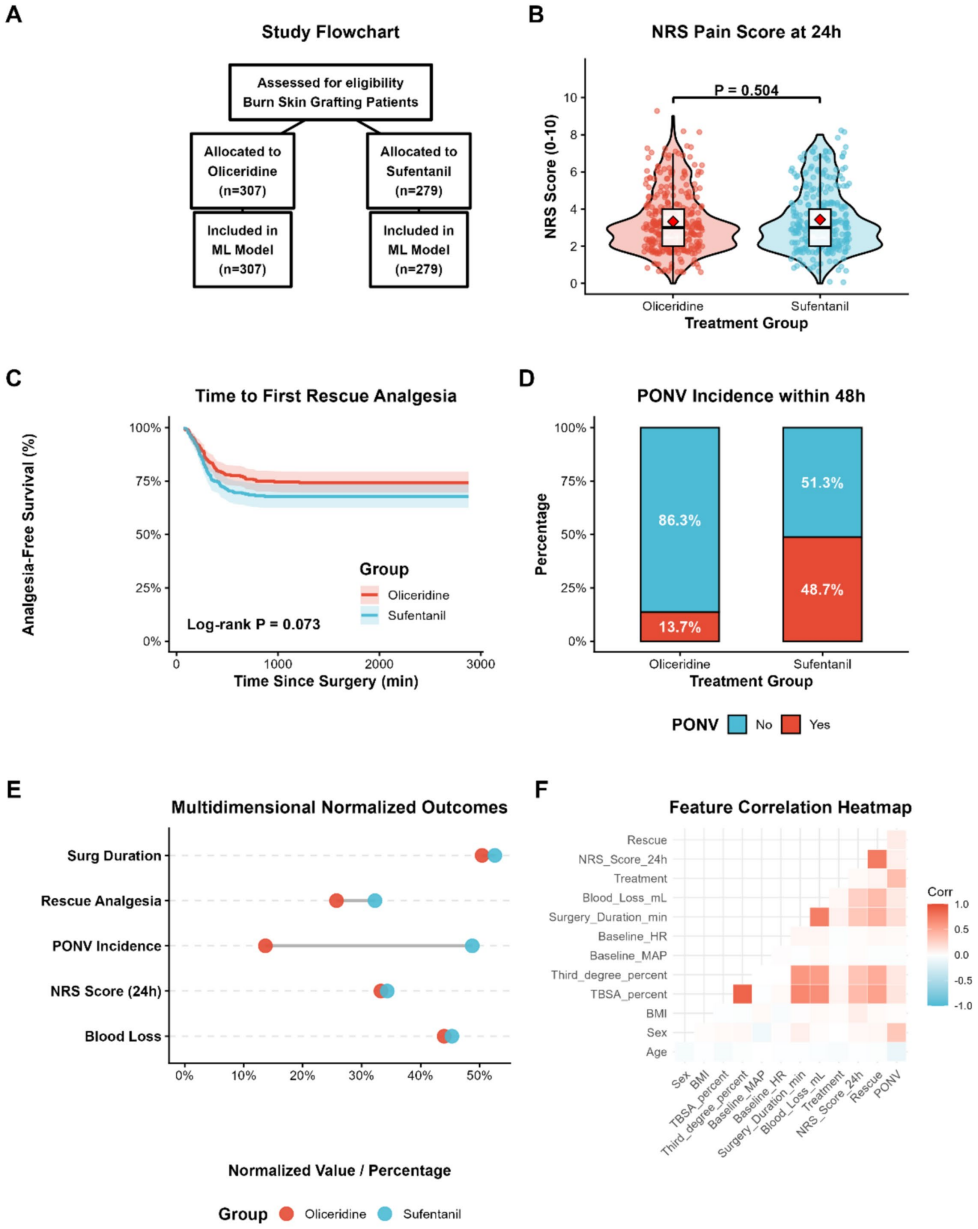
Study flowchart and comparative baseline clinical outcomes. **(A)** CONSORT flow diagram detailing the assessment, allocation, and inclusion of burn skin grafting patients into the machine learning models. **(B)** Violin plots superimposed with boxplots showing the distribution of the 24 h numeric rating scale (NRS) pain scores. The red diamond indicates the mean value. **(C)** Kaplan–Meier curves for the time to first rescue analgesia, with the shaded area representing the 95% confidence interval. **(D)** Stacked bar charts comparing the overall incidence of postoperative nausea and vomiting (PONV) within 48 h between the two cohorts. **(E)** Multidimensional normalized dumbbell plot illustrating the comparable predictive efficacy but distinct predictive safety profiles between Oliceridine and Sufentanil. **(F)** Spearman correlation heatmap of the input features to screen for multicollinearity prior to model training.

A total of 586 patients undergoing burn skin grafting were included in this study and allocated to either the Oliceridine group or the Sufentanil group (Figure 1A). Baseline demographic and perioperative factors depicted in Table 1 were carefully balanced in the two groups. Variables such as age, gender distribution, BMI, and ASA physical status did not indicate significant intergroup differences. Furthermore, surgical parameters (such as TBSA burned, third-degree burn area, and surgery duration) were highly comparable, establishing a robust foundation for minimizing confounding bias in subsequent outcome evaluations.

**Table 1:** Baseline demographic, perioperative characteristics, and clinical outcomes of the study cohort.

Clinical indicators (variables)	Total cohort (*N* = 586)	Oliceridine group (*n* = 307)	Sufentanil group (*n* = 279)	*p* value
Demographics				
Age (years), mean ± SD	45.3 ± 12.0	45.8 ± 12.2	44.8 ± 11.7	0.308
Gender (women), *n* (%)	240 (41.0%)	126 (41.0%)	114 (40.9%)	>0.99
BMI (kg/m ^2^), mean ± SD	24.2 ± 3.5	24.1 ± 3.5	24.3 ± 3.4	0.335
ASA physical status, *n* (%)				0.932
Grade I	161 (27.5%)	83 (27.0%)	78 (28.0%)	
Grade II	303 (51.7%)	161 (52.4%)	142 (50.9%)	
Grade III	122 (20.8%)	63 (20.5%)	59 (21.1%)	
Burn and surgery characteristics				
TBSA burned (%), median [IQR]	28.0 [20.0–36.0]	27.0 [20.0–35.0]	29.0 [21.0–38.0]	0.092
Third-degree burn area (%), median [IQR]	12.0 [9.0–16.0]	12.0 [9.0–16.0]	12.0 [9.0–17.0]	0.231
Baseline MAP (mmHg), mean ± SD	87.7 ± 9.8	87.8 ± 9.5	87.7 ± 10.2	0.885
Baseline HR (beats/min), mean ± SD	81.9 ± 10.8	82.2 ± 11.0	81.6 ± 10.6	0.478
Duration of surgery (min), mean ± SD	112.1 ± 29.3	109.9 ± 29.5	114.6 ± 29.0	0.051
Intraoperative blood loss (mL), Median [IQR]	320.0 [270.0–380.0]	320.0 [270.0–370.0]	320.0 [270.0–400.0]	0.304
Clinical outcomes (target labels)				
NRS pain score at 24 h (points), median (IQR)	3.0 [2.0–4.0]	3.0 [2.0–4.0]	3.0 [2.0–4.0]	0.504
Time to first rescue analgesia (min) < sup > a</sup>, median [IQR]	273.0 [188.0–374.0]	274.0 [184.5–387.5]	265.5 [193.2–343.5]	0.558
Rescue analgesia required within 48 h, *n* (%)	169 (28.8%)	79 (25.7%)	90 (32.3%)	0.099
PONV within 48 h, *n* (%)	178 (30.4%)	42 (13.7%)	136 (48.7%)	<0.001*

Normally distributed continuous variables are presented as mean ± standard deviation (SD) and compared using the independent samples t-test. Non-normally distributed continuous variables are presented as median [interquartile range, IQR] and compared using the Mann–Whitney U test. Categorical variables are presented as frequencies and percentages (*n*, %) and compared using the Pearson’s Chi-square test. ^a^Calculated only among patients who required rescue analgesia (event positive). *Indicates statistical significance (*p* < 0.05). BMI, Body mass index; ASA, American Society of Anesthesiologists; TBSA, Total body surface area; MAP, Mean arterial pressure; HR, Heart rate; NRS, Numeric rating scale; PONV, Postoperative nausea and vomiting.

The two opioid regimens demonstrated comparable clinical outcomes with regard to postoperative analgesic efficacy in this observational cohort. The median NRS pain scores at 24 h postoperatively were identical between the groups. Additionally, there was no significant difference in the proportion of patients requiring rescue analgesia within 48 h. This comparable outcome was also performed using the Kaplan–Meier survival analysis of the time to first rescue analgesia (Figure 1C), revealing no statistically significant separation between the survival curves, indicating a comparable duration of pain relief.

On the other hand, a high level of disparity in safety profiles was seen. The proportion of PONV in the first 48 h was significantly reduced in the Oliceridine group in relation to the Sufentanil group (Figure 1D). This unique safety benefit was clearly demonstrated in the multidimensional normalized outcome plot (Figure 1E). Parameters including surgery duration, intraoperative blood loss, and pain scores showed high similarity between the two groups, whereas the PONV incidence diverged substantially, highlighting that Oliceridine was associated with a significantly lower predicted risk of opioid-related adverse events while maintaining comparable predicted primary analgesic outcomes within this cohort.

Spearman correlation in form of a heatmap (Figure 1F) was performed before the development of ML models to assess the correlation between variables. In the identified correlation matrix, intrinsic clinical associations were identified, including (1) total burn area and duration of surgery (*r* = 0.69), and (2) 24-h pain and the need for rescue analgesia (*r* = 0.76). Notably, PONV incidence was positively correlated with the assigned treatment group (*r* = 0.26), whereas only a weak positive correlation was observed with analgesic outcomes. Among the main predictive factors, no severe multicollinearity that would preclude ML implementation was detected, supporting the appropriateness of the dataset for algorithm training.

### 3.2 Development and evaluation of efficacy prediction models

**Figure 2:**
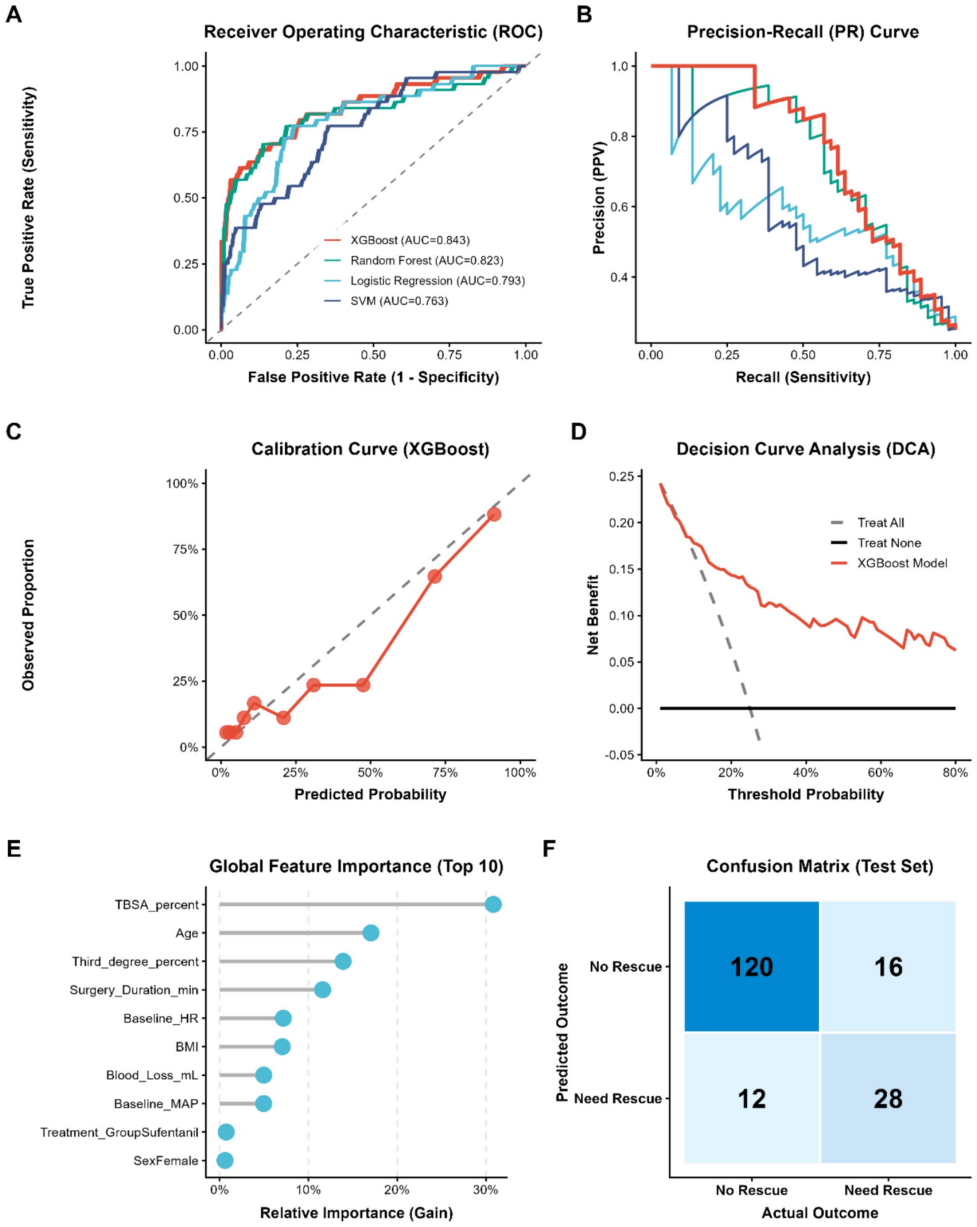
Performance evaluation of machine learning models predicting the need for postoperative rescue analgesia. **(A)** Receiver operating characteristic (ROC) curves of four distinct algorithms. The XGBoost model demonstrated the highest predictive discriminability. **(B)** Precision-recall (PR) curves demonstrating the robustness of XGBoost in handling clinical data. **(C)** Calibration curve of the XGBoost model, indicating a high concordance between predicted probabilities and observed proportions. **(D)** Decision curve analysis (DCA) showing that the XGBoost model provides a superior net clinical benefit across a wide range of threshold probabilities. **(E)** Global feature importance matrix derived from the XGBoost model (Gain metric). Note the negligible contribution of the “Treatment Group,” aligns with the comparable predictive associations for analgesia observed in this cohort. **(F)** Confusion matrix of the XGBoost model on the independent test set.

To systematically identify patients at high risk of inadequate pain relief requiring rescue analgesia, four ML algorithms were trained and validated. Figure 2A shows that the highest predictive performance recorded using the eXtreme Gradient Boosting (XGBoost) model, which has the highest Area Under Receiver Operating Characteristic Curve (AUC). This substantially outperformed the traditional LR model (AUC = 0.793) and other algorithms, including the RF (AUC = 0.823) and SVM (AUC = 0.763), suggesting that the tree-based ensemble method effectively captured complex, non-linear interactions among clinical variables that could have been missed in the linear models. Similar superiority was consistently observed in the precision-recall curve (Figure 2B), where XGBoost maintained higher positive predictive values across varying recall thresholds.

The XGBoost model was further confirmed as reliable for use in real-world clinical settings. Strong coherence between the predicted probabilities and the observed proportions of rescue analgesia requirements (calibration curve, Figure 2C) not only indicated the absence of overestimation or underestimation of risk but also showed that the risk was well-calibrated. In addition, the clinical utility of the model was validated using the decision curve analysis (Figure 2D). Across an extended spectrum of threshold probabilities (up to 80%), the use of XGBoost model to inform clinical decisions produced a consistently greater net benefit than default strategies of treating all patients or treating none.

A critical step in interpreting the XGBoost model examined the global feature importance of the native Gain measure (Figure 2E). The most common predictor (Gain = 30.8) was TBSA burned followed by age (17.1), third-degree burn area (13.9), and surgery duration (11.6). Interestingly, the kind of opioid that was given (Sufentanil versus Oliceridine) was at the lowest end of the list of predictive characteristics, adding 0.7% to the decision-making process in the model. This algorithmic finding perfectly mirrors the univariate analyses presented in Table 1 and Figure 1, providing data-driven evidence that the predicted need for rescue analgesia is comparable between Oliceridine and Sufentanil in this cohort. The confusion matrix for the independent test set (Figure 2F) showed a fairly high true-negative and a reasonable true-positive rate, which suggests that the model has strong discriminative ability on unseen clinical data.

### 3.3 SHAP interpretability analysis of the efficacy model

**Figure 3:**
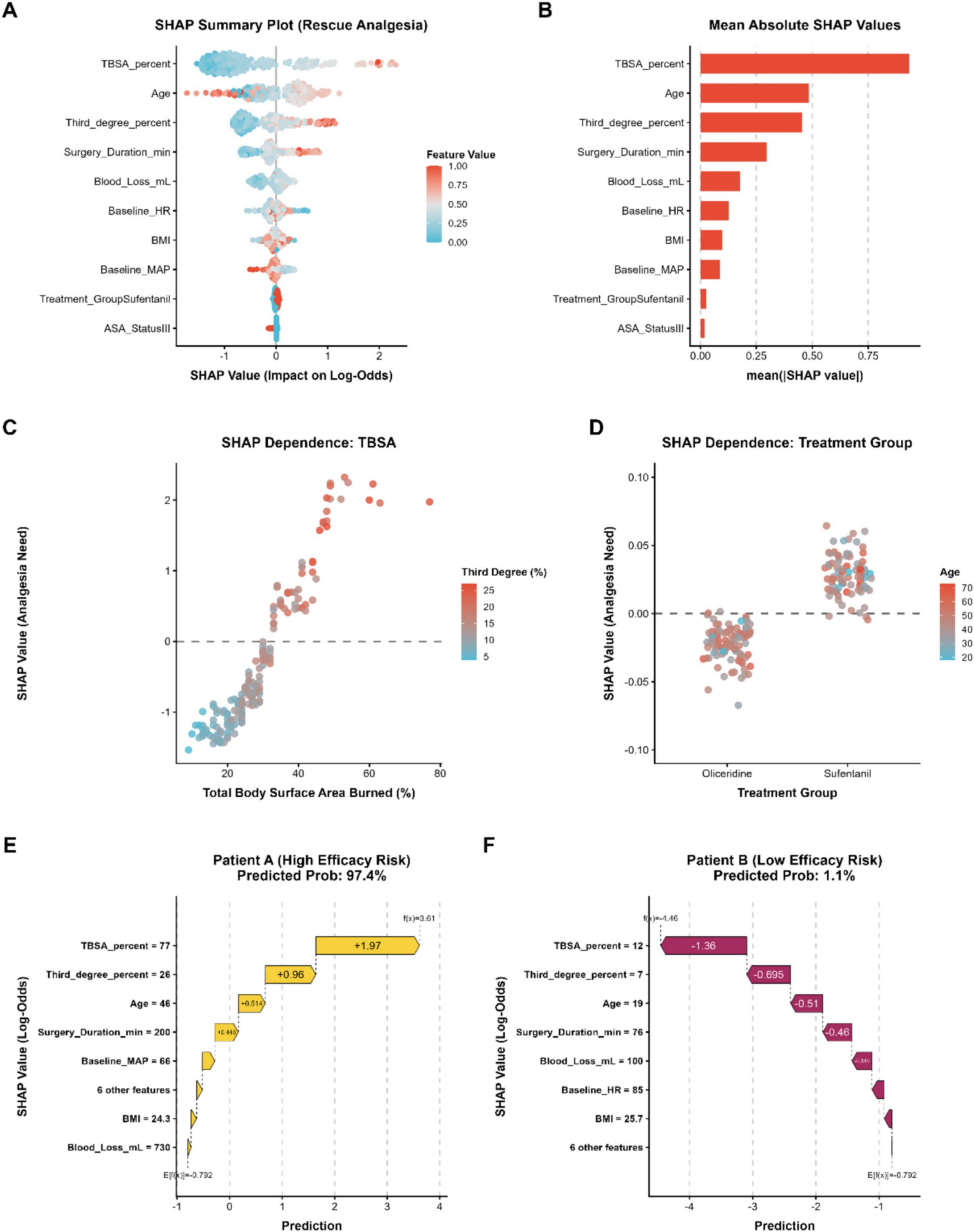
Shapley additive explanations (SHAP) interpretability analysis for the analgesic efficacy model. **(A)** SHAP summary beeswarm plot illustrating the global impact of features on predicting rescue analgesia. **(B)** Bar plot of mean absolute SHAP values ranking feature importance. **(C)** SHAP dependence plot for total body surface area (TBSA), revealing a non-linear risk escalation. **(D)** SHAP dependence plot of the treatment group. The SHAP values for both opioids clustered tightly around the zero baseline, aligning with their comparable predictive analgesic profiles. **(E)** SHAP waterfall plot for a high-risk patient (Patient A), showing how extensive burn area and prolonged surgery drove the risk log-odds. **(F)** SHAP waterfall plot for a low-risk patient (Patient B).

Following the establishment of the XGBoost efficacy model, SHAP analysis was employed to demystify the algorithmic decision-making process. These global feature attributions are depicted in Figures 3A,B. The average absolute SHAP values showed that TBSA burned was by far the most significant predictor (mean |SHAP| = 0.935), followed by patient age (0.485), third-degree burn area (0.456), and surgery duration (0.297). The administered opioid (Sufentanil versus Oliceridine), in its turn, had an insignificant effect (mean |SHAP| = 0.025) and ranked near the bottom of the clinical features. The beeswarm plot (Figure 3A) shows that SHAP values for the treatment group clustered around the zero baseline, implying that the drug choice was essentially directionless, that is, not exerting any directional influence on the predicted likelihood of needing rescue analgesia.

The feature dependence plots provided further insight into non-linear clinical thresholds. In the case of TBSA (Figure 3C), the log-odds of rescue analgesia quickly rose exponentially due to the size of the area burned, and the critical point was reached when the burned area was more than some 30% of the total. More to the point, the treatment assignment dependence plot (Figure 3D) had an extremely low, micro-scale variation (only between −0.10 and 0.10), symmetrically distributed around the zero line. This minor variation was stable among different age groups, further indicating a comparable predictive performance between Oliceridine and Sufentanil across ages without significant modeled bias.

SHAP waterfall plots indicated the detailed risk architecture of a particular patient archetype at the level of the individual one. Figure 3E shows that extensive TBSA (+1.97) and third-degree burn area (+0.96) were identified as the strongest predictive contributors in a patient with a high probability of requiring rescue analgesia (97.4% predicted rescue analgesia). Conversely, a low-risk patient with a predicted probability of 1.1% (Figure 3F) was mainly characterized by a small burn (−1.36). Notably, the treatment variable was not included in the top influential drivers in either case, demonstrating a predictive association that aligns with the hypothesis of comparable analgesic requirements between the two regimens.

### 3.4 Development and evaluation of safety prediction models for PONV

**Figure 4:**
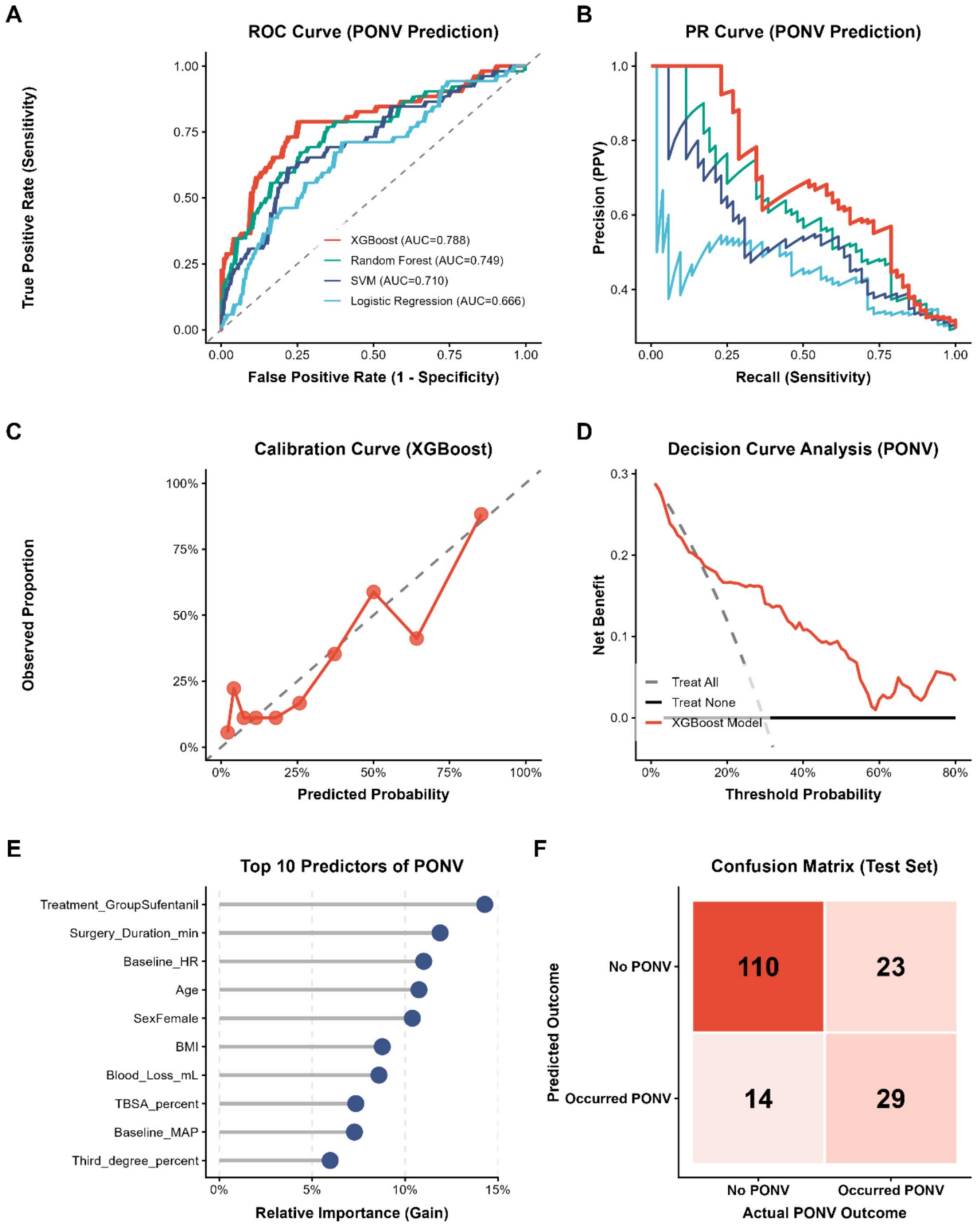
Performance evaluation of machine learning models predicting postoperative nausea and vomiting (PONV). **(A)** ROC curves highlighting the superior predictive capacity of XGBoost over traditional logistic regression in capturing non-linear safety profiles. **(B)** PR curves for PONV prediction. **(C)** Calibration plot of the best-performing XGBoost model. **(D)** DCA demonstrating the clinical utility of utilizing XGBoost to identify patients requiring prophylactic antiemetics. **(E)** Top 10 predictors of PONV ranked using XGBoost gain. **(F)** Confusion matrix showing the accurate classification of actual PONV occurrences.

While the efficacy evaluation suggested comparable predictive associations, determining the precise determinants of opioid-related adverse events remained crucial. As such, predictive models were formulated to predict the occurrence of PONV in 48 h. Figure 4A shows that the XGBoost model in this case also performed better in the discriminative capacity, with an AUC of 0.788. This was significantly higher than the performance of the conventional LR (AUC = 0.666), SVM (AUC = 0.710), and the RF (AUC = 0.749). The distinct performance gap (ΔAUC = 0.122 versus logistic regression) suggests that PONV risk is likely driven by complex, non-linear feature interactions rather than simple linear additive effects. Such predictive merit was evident throughout the precision-recall curve (Figure 4B), demonstrating that XGBoost was highly robust in identifying positive PONV cases present in an imbalanced clinical data set.

Clinical utility and model calibration were then compared. Figure 4C shows the calibration curve of the XGBoost model, demonstrating that it satisfactorily predicted probabilities and observed PONV incidences, especially in the clinically significant mid-to-low risk ranges, achieving a Brier score of 0.182. For transparency regarding class balance, the PONV positive-event counts were 125 in the training set (*n* = 410) and 53 in the test set (*n* = 176), ensuring that the predictive capability was evaluated on a representatively imbalanced cohort (overall PONV incidence was approximately 30.4%). The results of the decision curve analysis (Figure 4D) further supported that applying the XGBoost model in decision-making for prophylactic antiemetics resulted in a net clinical benefit sustained over a large range of threshold probabilities (up to 80%) compared to the treatment-all or treatment-none approaches.

Upon assessing the importance (Gain) of the global features (PONV) (Figure 4E), a paradigm shift was observed. Overall, in contrast to the efficacy model, in which the chosen opioid was insignificantly high in the risk of PONV, administration of Sufentanil emerged as the major predictor of PONV (Gain = 14.3%). The duration of the surgery was identified as the second risk factor (11.9%), and patient gender (women) was also among the influential risk factors (10.4%). This striking algorithmic contrast effectively decouples safety from efficacy: although substituting Sufentanil with Oliceridine appears to have minimal impact on pain relief, and the substitution constitutes the most decisive modifiable factor in mitigating PONV. A realistic incidence distribution (detecting 29 true positive cases) was also shown in the corresponding confusion matrix on the hold-out test set (Figure 4F), further supporting the predictive consistency of the model in this specific clinical cohort.

### 3.5 SHAP interpretability analysis decoding the safety advantages of Oliceridine

**Figure 5:**
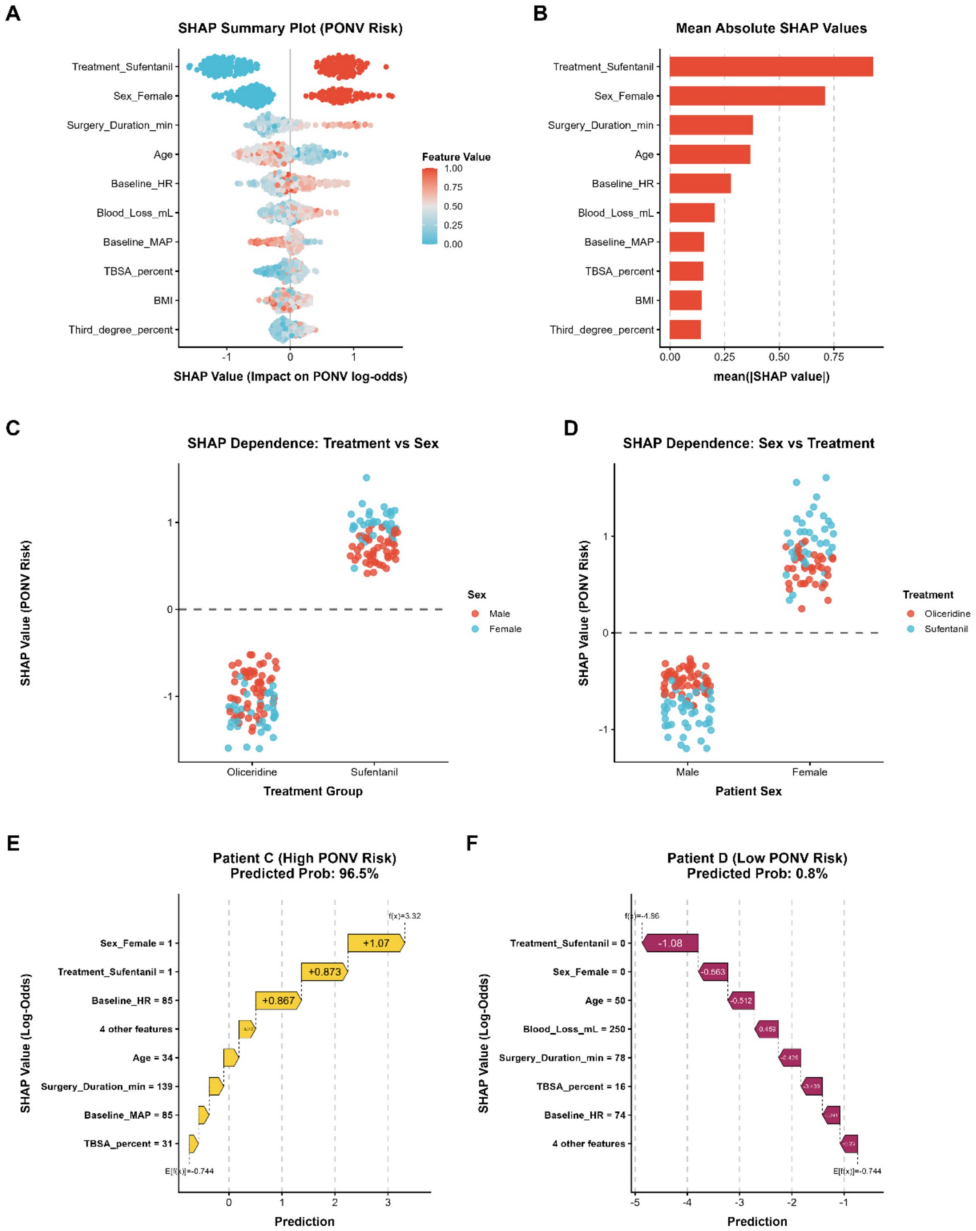
SHAP interpretability analysis dissecting the safety advantages of Oliceridine. **(A)** SHAP summary plot for PONV risk. **(B)** Mean absolute SHAP values emphasizing Sufentanil and women as the primary catalysts for PONV. **(C)** SHAP dependence plot illustrating the profound interaction between opioid choice and gender; women receiving Sufentanil faced exponentially higher PONV risks. **(D)** Alternate perspective of the gender-based-treatment interaction. **(E)** SHAP waterfall plot of a high-risk patient (Patient C), elucidating how Sufentanil administration exacerbated the risk. **(F)** SHAP waterfall plot of a low-risk patient (Patient D), visualizing the protective effect (negative SHAP value) of choosing Oliceridine.

Building upon the robust predictive performance of the XGBoost PONV model, SHAP analysis was used to decode the black-box predictive patterns associated with postoperative safety outcomes. In striking alignment with the global Gain measure, the average absolute SHAPs (Figure 5B) unambiguously defined that exact opioid treatment (mean |SHAP| = 0.810) and the gender of the patient (mean |SHAP| = 0.687) were the most significant predictors of PONV risk. The SHAP summary beeswarm plot (Figure 5A) showed a unitary monotonic main effect of the opioid choice: patients on Sufentanil (red dots) were all clustered at the right side above zero baseline showing a strong positive drive to vomiting, and on Oliceridine (blue dots), all were positioned on the left side below the zero baseline, which once again demonstrates significant protection against vomiting.

To further explore the internal decision-making patterns of the algorithm, SHAP dependence plots (Figures 5C,D) were built to map the modeled relationships among complex features. The analysis revealed a notable modeled interaction pattern between patient sex and opioid selection within the XGBoost framework (Figure 5D). Although the SHAP values showed low levels of PONV risk in male patients and remained consistently suppressed below zero despite the administered opioid type, the risk pattern was markedly different in female patients. There was a high-risk group represented with women who received Sufentanil, in which the SHAP values mathematically accumulated to peak levels (exceeding 1.0). In the model’s feature space, instances corresponding to Oliceridine lacked the elevated SHAP values seen with Sufentanil among women, resulting in mathematical output scores equivalent to the male patient baseline. This graphically illustrated a purely algorithmic interaction where the combined feature of “women” and “Oliceridine” did not trigger the mathematical risk penalty observed with “Sufentanil,” and should not be interpreted as a biological effect modification.

The clinical implications of these algorithmic findings are vividly demonstrated at the individual patient level through SHAP waterfall plots. In a female patient with a high risk of PONV that was predicted at 96.5% (Figure 5E, Patient C), the interaction between women (equal to 1.07) and Sufentanil administration (equal to 0.873) were the initial predictive risk factors and contributed to a steep increase in the predicted log-odds of the patient. On the other hand, in a low-risk patient where the probability of this event was estimated at 0.8% (Figure 5F, Patient D), the biggest protective pull (−1.08) was observed with the absence of Sufentanil use (such as Oliceridine administration), which safely positioned the patient in the safe zone. Algorithmically, these individualized SHAP value distributions highlight that the selection of Oliceridine over Sufentanil was consistently associated with a reduced predicted risk of PONV while maintaining similar importance profiles for primary analgesic outcomes.

### 3.6 Exploratory visualization and algorithmic risk stratification

**Figure 6:**
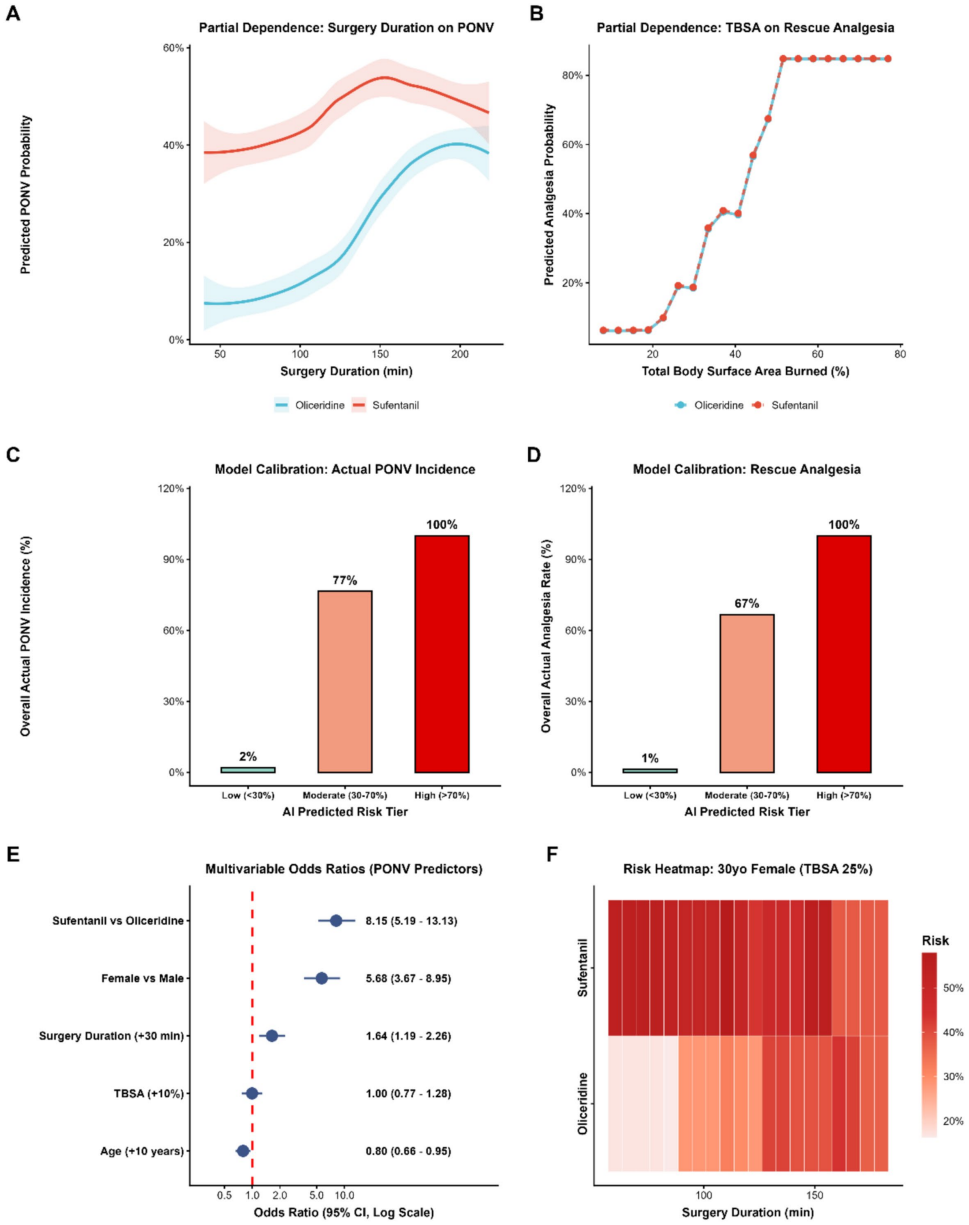
Exploratory visualization, partial dependence, and algorithmic risk stratification derived from the XGBoost framework. **(A)** Partial dependence plot (PDP) showing the marginal effect of surgery duration on predicted PONV probability between the two opioid groups. **(B)** PDP mapping the relationship between TBSA and the predicted need for rescue analgesia, showing intertwined identical trajectories for both drugs. **(C)** Clinical stratification of actual PONV incidence based on AI-predicted risk tertiles. **(D)** Clinical stratification for actual rescue analgesia requirements. **(E)** Multivariable forest plot of odds ratios (log scale) for classical clinical reference. **(F)** High-order interaction risk heatmap. This panel projects the dynamic PONV probability for a representative patient profile (30-year-old female, 25% TBSA) generated using the XGBoost model, illustrating the diverging risk trajectories between opioid choices across continuous surgery durations.

To visually represent the internal logic of the complex ML algorithms, the predictive models were mapped into exploratory visual frameworks. Partial dependence plots (PDP) were produced in order to represent the marginal impacts of the continuous variables on clinical outcomes. In the prediction of PONV (Figure 6A), the longer the surgery time the less predictable was the probability of PONV. Interestingly, the probability curve of Sufentanil (red line) remained steadily at a higher level of likelihood than Oliceridine (blue line) across all surgical periods, implying that the safety of Oliceridine was maintained even during long-term operations. Comparatively, the PDP of rescue analgesia (Figure 6B) showed completely overlapping curves of the two opioids as the total body surface area (TBSA) increased, which was consistent with the comparable predicted need for rescue analgesia.

The ongoing forecasted likelihoods of the XGBoost models were also stratified into sensible risk tertiles to assess clinical calibration. Both PONV and rescue analgesia across risk stratification levels– Low (<30%), Moderate (30–70%), and High (>70%)–demonstrated adequate calibration and exhibited a stepwise progression across the predicted risk tiers (Figures 6C,D). For example, the actual PONV incidence accurately escalated from 0% in the low-risk cohort to 100% in the high-risk cohort (Figure 6C). This monotonic calibration indicates that the model was reasonably calibrated within this internal retrospective dataset, generating hypotheses for future potential applications in patient triage for prophylactic interventions.

To compare the ML results with the traditional statistical paradigms, a multivariate LR forest plot was constructed (Figure 6E). In agreement with the SHAP analysis, Sufentanil use (OR = 8.15, 95% CI: 5.19–13.13) and women (OR = 5.68, 95% CI: 3.67–8.95) were the most effective independent participants to predict PONV. Lastly, a high-order interaction risk heatmap (Figure 6F) was generated to replicate a representative high-risk profile (a 30-year-old female patient with one-fourth of the total area covered by TBSA). The heatmap provides a direct clinical visualization: although the predicted likelihood of PONV escalates with longer surgery under Sufentanil, substituting it with Oliceridine in the model was associated with a lower predicted risk trajectory, serving as a hypothesis-generating visual aid for individualized opioid choices.

## 4 Discussion

Postoperative pain management following burn skin grafting remains a critical clinical challenge, necessitating an optimal balance between profound analgesia and the mitigation of opioid-related adverse events such as postoperative nausea and vomiting (PONV) (21, 22). This research employed ML and SHAP interpretability to show that the novel biased mu-opioid receptor agonist, Oliceridine, exhibits a comparable predictive analgesic profile to Sufentanil while being associated with a lower predicted risk of PONV. These XGBoost models demonstrated promising predictive capabilities; however, the metrics derived from the 7:3 hold-out test set may be interpreted as optimistic estimates due to the single data split. Furthermore, these models structurally disaggregated the predictors of efficacy and safety. This mathematical separation provides a foundational framework for generating preliminary risk stratification hypotheses.

This observation of comparable predictive analgesic profiles is consistent with preliminary phase III studies of Oliceridine, which reported similar pain management trajectories to morphine during both the hard tissue and soft tissue surgeries. This knowledge is applied in the context of serious burn surgeries in the ML analysis. Patient-intrinsic predictors, which are total body surface area burned and duration of surgery, were the absolute dominant predictors in the efficacy prediction model, and the specific opioid given had a minimal impact in both the XGBoost Gain metric and SHAP analysis. With the SHAP dependence plot for treatment assignment remaining virtually flat and centered around zero, the model illustrates a lack of strong predictive association between the specific opioid choice and the likelihood of insufficient analgesia in this algorithm. This evidence-based finding may encourage clinicians that the choice of Oliceridine within this modeled framework is associated with improved safety predictions and showed similar predicted analgesic outcomes for treating acute pain (23, 24).

Conversely, the safety model revealed a stark paradigm shift, identifying the choice of opioid as the paramount predictor of PONV. This study found that in the multivariate analysis that the risk of PONV with Sufentanil was much greater, which was consistent with the existing literature, stating that traditional opioids unselectively activate both G-protein and *β*-arrestin pathways, thereby triggering emetic centers (25, 26). Interestingly, the analysis of the SHAP interaction revealed a specific modeled pattern: female patients, who represent a well-known high-risk category in the Apfel score, exhibited a substantially heightened predicted risk trajectory for PONV when the model incorporated the administration of Sufentanil. Within the model’s algorithmic architecture, a purely mathematical observation emerged: the computed penalty in predicted PONV log-odds associated with women was virtually absent when Oliceridine was the assigned feature, resulting in output values similar to the male patient baseline. However, given the observational nature of this study and the lack of robust control for critical perioperative confounders (such as volatile anesthetic exposure, standardized antiemetic prophylaxis, and cumulative opioid dosing), this modeled treatment-based on gender/sex pattern remains strictly an algorithmic observation and necessitates prospective clinical validation to determine any true biological interaction. When comparing the native XGBoost Gain plot and the SHAP mean absolute value plot, the reviewer or reader can observe a minor discrepancy between the internal ranking of secondary features (such as surgery duration and gender). This minor discrepancy is methodologically well-founded and reflects the fact that Gain and SHAP capture different, yet complementary, aspects of feature importance. While the native Gain metric measures the overall reduction in entropy during global tree splitting—which inherently favors continuous variables with multiple potential split points (27, 28)—SHAP quantifies the direct, instance-level magnitude of impact on the predicted log-odds, thereby appropriately elevating the clinical relevance of binary demographic factors like gender in localized predictions (29, 30). Presenting both metrics provides a more holistic and complementary understanding of the model’s architecture.

Despite yielding robust predictive algorithms, this study has several notable limitations inherent to its retrospective observational design. First, the treatment allocation between Oliceridine and Sufentanil was not randomized but rather determined by clinician preference and temporal drug availability. Consequently, significant unmeasured confounders—such as variations in intraoperative opioid dosing equivalents, standardized antiemetic prophylaxis protocols, and perioperative adjunct analgesics—were not controlled through the propensity score matching or rigorous causal inference frameworks. Both primary outcomes—the decision to administer rescue analgesia and the pharmacological intervention for PONV—were inherently practice-dependent. Consequently, substantial residual variability was driven by subjective institutional prescribing thresholds, nursing practices, and clinician preferences. Therefore, it is critical to emphasize that the model’s predictions partially reflect institutional behaviors rather than purely physiological responses. The identified relationships represent algorithmic associations that are heavily influenced by this clinical variability. Future prospective, double-blind randomized controlled trials are essential to validate these hypothesis-generating predictive findings. Second, the study models mainly used the structured preoperative and intraoperative electronic health record data, with no dynamic perioperative physiological streams (such as continuous intraoperative electroencephalograph, EEG; nociception indices, and intraoperative fluctuations in opioid consumption), which could further improve predictive accuracy. Third, the validation strategy was restricted to a single 70/30 data split. Even with internal cross-validation for hyperparameter tuning, this single-split approach in a dataset of this size remains vulnerable to optimism, limiting the robustness of model performance interpretation. Therefore, explicit external validation using independent, multicenter datasets—or leveraging large, publicly available clinical databases such as the medical information mart for intensive care IV (MIMIC-IV), a strategy that has proven highly valuable in validating critical care prediction models (31, 32)—is required to confirm true predictive reliability and ensure generalizability across varying surgical demographics and institutional protocols prior to any clinical deployment. Furthermore, while this study focused specifically on analgesic efficacy and safety, it contributes to the expanding landscape of data-driven burn management, complementing existing literature that utilizes algorithmic risk stratification—such as nomograms for predicting intraoperative hypothermia during escharotomy (33)—and aligning with the broader trend of integrating advanced therapies into comprehensive burn care pathways (34, 35).

## 5 Conclusion

In conclusion, this study successfully developed robust ML models to predict postoperative analgesic efficacy and safety in patients undergoing burn skin grafting. These findings, powered by XGBoost and SHAP interpretability, suggest that Oliceridine showed similar predicted analgesic outcomes to Sufentanil, as treatment choice had negligible impact on predicting rescue analgesia needs in this model. Conversely, the use of Oliceridine instead of Sufentanil was associated with a substantially lower predicted risk of PONV-, particularly exhibiting a blunted modeled risk pattern in female patients compared with the heightened risk trajectory associated with Sufentanil. These mathematical algorithms presently serve solely as exploratory, hypothesis-generating computational tools. Because the reported performance metrics should be viewed as optimistic estimates derived from a single data split, exhaustive prospective multicenter validation is required before these models can advance beyond their existing methodological proof-of-concept stage.

## Data Availability

The raw data supporting the conclusions of this article will be made available by the authors, without undue reservation.
